# 603. Determining Effect of Media and Disk Source on Reproducibility and Relationship between Broth Microdilution and Disk Diffusion Testing of Cefiderocol

**DOI:** 10.1093/ofid/ofad500.670

**Published:** 2023-11-27

**Authors:** Miki Takemura, Mizue Saiki, Hidenori Yamashiro, Naomi Anan, Boudewijn L DeJonge, Christine M Slover, Christopher M Longshaw, Anne Henriksen, Yoshinori Yamano

**Affiliations:** Shionogi & Co., Ltd, Toyonaka, Osaka, Japan; Shionogi & Co., Ltd., Toyonaka, Osaka, Japan; Shionogi & Co., Ltd., Toyonaka, Osaka, Japan; Shionogi & Co., Ltd., Toyonaka, Osaka, Japan; Shionogi Inc., Florham Park, New Jersey; Shionogi Inc., Florham Park, New Jersey; Shionogi B.V., London, England, United Kingdom; Shionogi BV, Copenhagen, Hovedstaden, Denmark; Shionogi & Co., Ltd., Toyonaka, Osaka, Japan

## Abstract

**Background:**

Cefiderocol (CFDC) is a siderophore cephalosporin with broad activity against Gram-negative bacteria. Discrepancies in susceptibility testing for CFDC have been reported. In this study susceptibility testing was performed using broth and agar medium and disks from various manufacturers to evaluate the reproducibility across the different sources and determine the relationship between MIC and inhibition zone.

**Methods:**

12 Enterobacterales, 12 *P. aeruginosa*, 12 *A. baumannii* with *in vivo* efficacy results in the murine thigh infection model under humanized PK of CFDC were evaluated. MICs were determined (10 replicates per media per isolate per day) using broth microdilution with iron-depleted cation-adjusted Mueller-Hinton broth (ID-CAMHB) prepared from BD BBL, BD Difco, Oxoid and Merck. The disk zone was determined (3 replicates per media/disk per isolate per day) with CFDC disks from Hardy and Liofilchem and Mueller Hinton Agar from BBL and bioMerieux. Testing was conducted for 3 days using the same inoculum for MIC and disk diffusion studies on each day. Susceptibility was interpreted according to 2023 CLSI breakpoints.

**Results:**

Good reproducibility of MIC was observed for the ID-CAMHB from each manufacturer for all isolates, except for 2 *A. baumannii* due to heavy trailing and skip. MIC variation was observed between ID-CAMHB from each manufacturer. Best categorical agreement with *in vivo* efficacy was observed for the MIC obtained with ID-CAMHB from BD BBL. Good reproducibility of disk inhibition zones were obtained for Enterobacterales and *P. aeruginosa* irrespective of the manufacturer of disks and agar medium. *A. baumannii* isolates with elevated MIC values frequently showed colonies in the inhibition zones, with more colonies appearing on BBL agar. This phenomenon was not reproducible, so larger variation of inner inhibition zones were observed with these isolates.

**Conclusion:**

MIC determinations were reproducible with each ID-CAMHB medium, but variability was observed across the different ID-CAHMB; MICs obtained with BD BBL ID-CAMHB showed best correlation with *in vivo* data. Good categorical agreement was observed for MIC in BD BBL and BD Difco medium and disk zone, despite variable inhibition zones observed for *A. baumannii*.

**Disclosures:**

**Miki Takemura, n/a**, Shionogi & Co., Ltd.: Stocks/Bonds **Mizue Saiki, Ph.D.**, Shionogi & Co., Ltd.: employee **Hidenori Yamashiro**, Shionogi & Co., Ltd.: employee **Naomi Anan, MSc**, Shionogi & Co., Ltd.: employee **Boudewijn L. DeJonge, PhD**, Shionogi Inc.: Employee **Christine M. Slover, PharmD**, Shionogi,INC: Employee **Christopher M. Longshaw, PhD**, Shionogi BV: Employee **Anne Henriksen, PhD**, Shionogi: Employee **Yoshinori Yamano, PhD**, Shionogi HQ: Employee

